# Blueberries Improve Abdominal Symptoms, Well-Being and Functioning in Patients with Functional Gastrointestinal Disorders

**DOI:** 10.3390/nu15102396

**Published:** 2023-05-20

**Authors:** Clive H. Wilder-Smith, Andrea Materna, Søren S. Olesen

**Affiliations:** 1 Brain-Gut Research Group, Gastroenterology Group Practice, 3011 Bern, Switzerland; 2 Mech-Sense, Department of Gastroenterology & Hepatology, Aalborg University Hospital, 5000 Aalborg, Denmark; soso@m.dk

**Keywords:** polyphenols, irritable bowel syndrome, functional dyspepsia, disorders of gut–brain interaction, visceral pain, nutraceutical, fructose

## Abstract

Blueberries beneficially modulate physiologic mechanisms relevant to the pathogenesis of functional gastrointestinal disorders (FGID). Forty-three patients with FGID received freeze-dried blueberries (equivalent to 180 g fresh blueberries) or sugar and energy-matched placebo in a double-blind, randomized, cross-over study. After 6 weeks of treatment, the differences in Gastrointestinal Clinical Rating Scale (GSRS) scores and abdominal symptom relief were compared as primary outcome measures. The quality of life and life functioning ratings (OQ45.2 questionnaire), Bristol stool scales, and fructose breath test results constituted secondary outcome measures. Blueberry treatment resulted in more patients with relevant abdominal symptom relief compared to placebo (53% vs. 30%, *p* = 0.03). Total and pain GSRS scores improved insignificantly (mean treatment differences [95% CI]: −3.4 [−7.4 to 0.6] (*p* = 0.09) and −1.0 [−2.2 to 0.1] (*p* = 0.08), respectively). OQ45.2 scores improved during blueberry treatment compared to placebo (treatment difference −3.2 [95% CI: −5.6 to −0], *p* = 0.01). Treatment effect differences for the further measures did not reach statistical significance. Blueberries relieved abdominal symptoms and improved general markers of well-being, quality of life, and life functioning more than placebo in patients with FGID. Consequently, the polyphenol and fiber components of blueberries exert broad beneficial effects separate from the sugars present in both treatments.

## 1. Introduction

Functional gastrointestinal disorders (FGID), such as irritable bowel syndrome (IBS) and functional dyspepsia (FD), rank amongst the most frequent causes of gastrointestinal symptoms, with a prevalence of between 10 and 15% in most populations [1]. The mechanisms underlying FGID remain unclear, but immune activation, nervous system sensitization, modulation of gut permeability, and changes in the enteric microbiome are recognized as interrelated components, all of which are affected by dietary factors [2,3,4,5,6,7,8]. The symptoms of FGID, recently termed disordered gut–brain interactions (DGBI) are multiple and variable and include abdominal pain or discomfort, changes in bowel patterns, signs of excessive fermentation, as well as extra-gastrointestinal manifestations [5]. The composition and metabolic activity of the enteric microbiota appear to play an important role in several of the implicated mechanisms, via nutrient metabolism and modulatory effects on the human host systems [8,9]. Conversely, dietary components have been shown to significantly influence the enteric microbiota, with downstream systemic and epigenetic effects in the host [10].

Polyphenols are amongst the most abundant plant metabolites and the most common antioxidants in our food. Blueberries (genus *Vaccinium* sect. *Cyanococcus)* are amongst the best studied polyphenol-rich fruit, and mechanistic and epidemiological studies have indicated a reduced risk of cardiovascular disease, type 2 diabetes, and death, as well as improved brain and possibly visual function with regular consumption [11,12,13,14,15]. Although blueberries have several potentially beneficial actions on mechanisms particularly pertinent to FGID, such as antioxidation, antiinflammation, membrane permeability reversal, and neuroprotection, we are not aware of any clinical studies in patients with FGID [12,14,16,17,18,19,20].

The aim of the current study was to probe the effects of blueberries given for a prolonged period on clinical symptoms and mechanistic laboratory measures relating to FGID. Fructose breath tests were included in the study to investigate the role of enteric microbiotic metabolism of fructose in the symptoms in patients with FGID.

Our study hypothesis was that consumption of blueberries would improve patients’ overall functioning and quality of life and thereby clinical outcome by decreasing symptoms and impact mechanistic biomarkers of FGID compared to a placebo with similar sugar composition but without dietary fiber and polyphenol content.

## 2. Materials and Methods

### 2.1. Design

**Figure 1:**
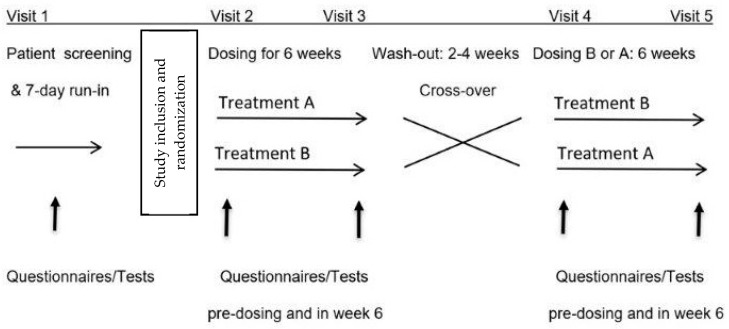
Study design.

Consecutive patients referred to our clinic with FGID were invited to participate in this prospective, randomized, double-blind, placebo-controlled, 2-arm cross-over and single-center study (Figure 1).

### 2.2. Patients

Selection criteria: Fifty-five successive male or female white patients of age 18–60 years and body mass index of 18.5–32.9 kg/m^2^ having IBS or FD or both according to the Rome 4 criteria as their major complaint were enrolled from the Gastroenterology Group Practice in Bern, Switzerland [2,21]. One author (CWS) assessed all patients clinically.

Exclusion criteria were evidence of other clinically significant diseases, as assessed by clinical history, blood and stool tests, ultrasound or CT imaging and endoscopy. Further exclusion criteria were colonoscopy, antibiotic or probiotic treatment within the two weeks before or during the study, planned dietary modifications (including polyphenol-rich fruit or vegetable smoothies, drinks or diets) or initiation of new medications during the study period, ongoing pregnancy or breast-feeding, and the inability to comprehend or contraindications to undergo the study procedures.

### 2.3. Study Procedures

A seven-day screening period with symptom observation was performed to assess eligibility for the study. If study inclusion and exclusion criteria were met and written informed consent was given, patients were randomized in equal proportions by the treatment-blinded study nurse to begin the study with either blueberries or placebo treatment in balanced blocks of 10 subjects, each generated using the website www.randomization.org. Each treatment period lasted six weeks, with subsequent cross-over to the alternate treatment after a washout period of two to four weeks without treatment.

Patients maintained their usual background diet throughout the study, avoiding the introduction of new dietary content and abstaining from additional polyphenol-rich foods sources, such as fruit or vegetable smoothies, drinks or supplements.

### 2.4. Questionnaires, Tests, and Biological Samples

At baseline, patients completed the standard Gastroenterology Group Practice symptom and Gastrointestinal Symptom Rating Scale (GSRS) questionnaires to assess demographics, GI and extra-GI symptoms, personal history, and dietary habits [22,23]. The Hospital Anxiety and Depression Scale (HADS), State-Trait Anxiety Inventory (STAI), Patient Health Questionnaire-15 (PHQ-15), and International Physical Activity Questionnaire (IPAQ) questionnaires were also completed [24,25,26,27].

During treatment periods, the tests listed below were performed within 4 weeks after study inclusion and before the first treatment period, before the start of the second treatment period after a minimum washout period of 14 days, and in the 6th week of each treatment period (Figure 1). The assessments were carried out in the same 60 min time window on the mornings of study days, under climate-controlled (20–23 °C) and quiet conditions in dedicated rooms in our practice, and after an initial 15 min rest. Patients arrived after an overnight fast and a standardized low-FODMAP diet on the previous day.

### 2.5. Primary Outcome Variables

The two primary outcome variables were clinical GI ratings, namely the GSRS scores validated in German and a global symptom rating statement based on FDA recommendations, and comprised of the following question: “How would you rate your abdominal signs or symptoms overall over the past 7 days?” [22,23,28] The Likert-scale-based possible responses were significantly relieved = +2, moderately relieved = +1, unchanged = 0, moderately worse = −1, and significantly worse = −2^28^. The GSRS scores and the numbers of responders defined by the global symptom relief question as having had either moderate or significant relief were compared after 6 weeks dosing with blueberries and placebo.

### 2.6. Secondary Outcome Variables

- Quality of life and areas of life functioning (symptoms, interpersonal problems, social role functioning) using the Outcome Questionnaire (OQ-45.2) scales in German, applying a cut-off score of >63 for clinically significant compromise [29,30].
- Bristol Stool Scale (BSS). The proportion of patients with normal stool consistency was compared.
- Fructose breath tests. Hydrogen and methane breath concentrations were measured before, 1 and 2 h following ingestion of 35 g fructose dissolved in 300 mL tap water (Quintron BreathTracker SC^®^, Quintron Instruments, Milwaukee, Brookfield, WI, USA). The following GI and extra-GI symptoms were scored hourly and rated for intensity (none = 0, mild = 1, intense = 2) concurrently with the collection of the breath samples: abdominal pain, arthralgia, bloating, borborygmi, diarrhea, diminished concentration, epigastric pain/heartburn, flatulence, fullness, headache, myalgia, nausea, and tiredness [5,31]. The fructose test was performed in accordance with previous studies [5,31].

Further detailed measurements of cognitive function, faecal collections for microbiome analysis, blood samples for metabolomics, and barrier permeability tests were accrued and will be reported in further publications.

### 2.7. Treatments and Blinding

Blueberry: 30 g of highbush freeze-dried blueberry powder produced from equal proportions of Tifblue^®^ and Rubel^®^ varieties and equivalent to 180 g fresh blueberries (395 kcal/100 g, total carbohydrates 93 g/100 g, total fiber 24 g/100 g, fructose 30 g/100 g, glucose 30 g/100 g, total phenolics 32 mg/g analyzed by the Folin-Ciocalteu method, anthocyanins 11.4 mg/g).

Placebo: 30 g energy-content-, color-, appearance-, and taste-matched blueberry powder placebo (362 kcal/100 g, total carbohydrates 90 g/100 g, total fiber: maltodextrin 22 g/100 g, dietary fiber: 1 g/100 g, fructose 37 g/100 g, glucose 35 g/100 g).

Both treatments were taken as two doses of 15 g powder daily, dissolved in 300 mL tap water and ingested within 30 min after breakfast and dinner meals. The blueberry and placebo powders were kindly supplied by the US Highbush Blueberry Council, USHBC, and conform with the powders used in several previous published trials.

The powder marked with the sequential randomization code was dispensed by the treatment-blinded study technician in individual dosage bags at the start of each treatment period and treatment compliance was ascertained by a dedicated weekly phone messaging system.

All study personnel and the patients remained blinded to the treatment administered until completion of the statistical analysis.

### 2.8. Statistics

The study was powered to detect a minimal difference between placebo and blueberry treatment of 10 points on the GSRS total score at the end of the two study periods (week 6). This corresponds approximately to the 30% symptom reduction considered to be clinically relevant assuming a baseline GSRS score of 30 [32,33]. Based on a standard deviation of 15, we determined that a study with 50 patients in a cross-over design was needed to provide a power of 90% (to allow for secondary endpoints), with the use of a two-sided significance level of 0.05 (alpha). Five extra patients were added as a safety measure, arriving at a total recruitment target of 55 patients [22].

All data were analyzed according to the intention-to-treat principle. Data are presented as means with standard deviations (SDs) or numbers (%) unless otherwise stated. For analysis of the primary endpoint, the GSRS scores at the end of the two treatment periods were compared using a linear mixed model including treatment regimen (blueberry vs. placebo), sequence, and treatment period as fixed effects and the patient (nested in the sequence of treatment periods) as a random effect. The retrieved model estimate was the mean difference between blueberry and placebo treatment reported with a 95% confidence interval. The assumption that the wash-out phase was long enough to rule out a carry-over effect and that no period effect was evident was also checked in the linear mixed model. Categorical data were analyzed using McNemar’s test. Secondary endpoints were analyzed using similar approaches as described for the primary endpoints. All the statistical analyses were performed using statistical software package STATA V.17.0 (StataCorp LP, College Station). *p*-values < 0.05 were considered statistically significant.

### 2.9. Ethics

All patients gave their written informed consent before study participation. The study was approved by the Cantonal Ethics Committee in Bern, approval number 2019-01593, was registered in ClinicalTrials.gov (NCT04824976), and performed according to the latest Declaration of Helsinki. All authors had access to the study data and reviewed and approved the final manuscript.

## 3. Results

**Figure 2:**
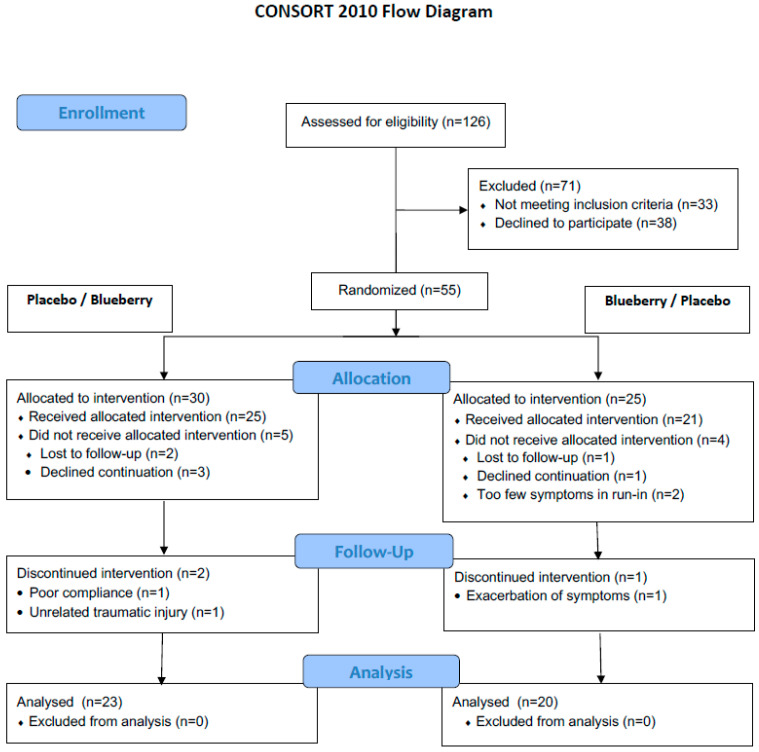
CONSORT study flowchart.

Of the 55 patients screened for the study, 43 were fully evaluable. The CONSORT patient flow and exclusion reasons are shown in Figure 2. Patient baseline characteristics are summarized in Table 1. There were no significant group differences in baseline characteristics, notably in gender, age or BMI distribution, type of FGID, dietary preferences, physical activity, and psychological questionnaire results.

### 3.1. Treatment Compliance

High compliance rates for treatment dosing were demonstrated by an average omission of 3.6 (4%) of the total 84 doses in treatment period 1 and of 5.6 (7%) of the total 84 doses in treatment period 2.

### 3.2. Primary Outcomes

There were no significant differences in total or subscale GSRS scores after 6 weeks of blueberry versus placebo treatment. Nonetheless, trends were seen in the differences between blueberry and placebo treatments in mean total GSRS scores (mean difference −3.4, 95% CI [−7.4 to 0.6], *p* = 0.09) and in GSRS pain subscale scores (mean difference −1.0, 95% CI [−2.2 to 0.1], *p* = 0.08) (Table 2). The remaining subscale GSRS scores also consistently showed lower scores with blueberry treatment vs. placebo but fell short of statistical significance (Table 2).

**Figure 3:**
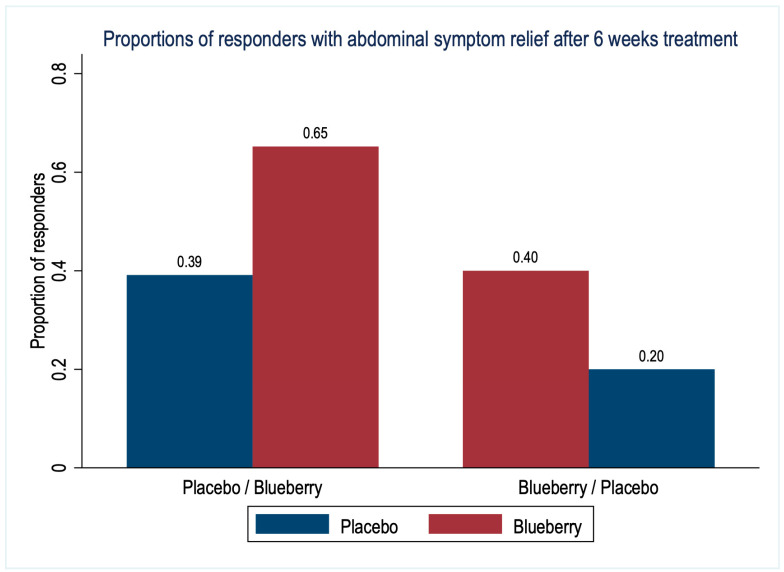
Proportions of responders with abdominal symptom relief after 6 weeks treatment with blueberries or placebo (n = 43 patients).

The proportions of responders based on abdominal symptom relief in the last week of each treatment period are shown in Figure 3. Overall, 23 (53%) patients reported relevant moderate or significant symptom relief after blueberry treatment compared to 13 (30%) patients after placebo treatment (difference 23%, 95% CI [1 to 46%], *p* = 0.03).

### 3.3. Secondary Outcomes

Stool consistency: The numbers of patients with Bristol Stool Scale scores reflecting diarrhea, normal stool consistency, and constipation after 6 weeks treatment were 7 (16%), 34 (79%), and 2 (5%) with blueberry and 12 (28%), 24 (56%), and 7 (16%) with placebo treatment (*p* = 0.10).

Breath tests: The effects of blueberry and placebo treatments on the mean AUC of breath hydrogen and methane concentrations over the first 2 h after the fructose challenge were not significantly different (Table 2). There was a significant carry-over effect for breath methane concentrations when blueberry treatment was given in the first period (*p* = 0.04).

The effects of both treatments on the reported GI and CNS symptoms during the fructose breath tests were not significantly different and there were no significant carry-over or period effects (Table 2).

### 3.4. Quality of Life and Life Functioning Outcome

**Figure 4:**
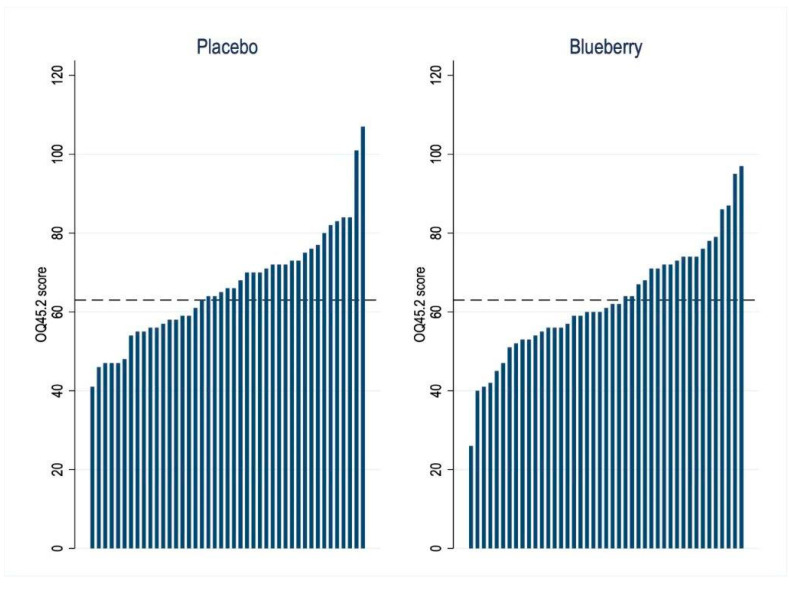
Waterfall figure of individual general well-being and functioning (OQ45.2) scores after 6 weeks of treatment with blueberries or placebo (n = 43 patients). The cut-off score > 63 indicates clinically significant compromise.

Blueberry treatment significantly improved, i.e., lowered, the mean OQ45.2 scores compared to placebo (difference −3.2, 95% CI [−5.6 to −0.7], *p* = 0.01). An OQ45.2 score of >63 (indicating clinically significant compromise) was evident in 19 (44%) patients after blueberry and in 25 (58%) patients after placebo treatment (difference 14%, 95% CI [1 to 28%], *p* = 0.03) (Figure 4).

### 3.5. Adverse Events

No serious adverse events occurred during the study. One patient developed a vesicular, pruritic generalized skin rash accompanied by a diminished ability to concentrate and constipation which lasted throughout the entire placebo-dosing period and was related to the colorant in the placebo preparation. Moderate nausea following placebo ingestion was noted in two patients in the first week of dosing.

## 4. Discussion

In patients with FGID, six weeks of treatment with blueberries significantly improved one of the two main primary outcomes, abdominal symptom relief, and the secondary outcome variable of overall well-being, quality of life, and life functioning compared to placebo treatment. The beneficial effects of blueberry treatment on symptom relief and the OQ45.2 imply improvement in important, interdependent areas of functioning, performance, and quality of life related to physical symptoms and mental health. The OQ45.2 measure is explicitly devised to track changes in these qualities over time [34]. This improvement is at least partly due to GI symptoms, as demonstrated by the significantly better abdominal symptom relief with blueberries. Depending on the sequence of treatments, between 40 and 65% of patients indicated abdominal symptom relief in the last week of blueberry treatment, compared to between 20 and 39% with placebo. The abdominal symptom relief was lower for both treatments in the second period (borderline effect), most likely explained by a carry-over beneficial effect from the treatment in the first period, i.e., from sugars in both treatments, or general improvements due to attention and care within the study. Changes in global abdominal symptom relief are likely to reflect the summation of symptom changes and their burden on life and functioning, which is not evident in specific, individual symptoms scores.

Differences in treatment effects on the individual GI symptoms assessed by the GSRS fell short of significance. There were trends to fewer patients with hard stool consistency and a reduction of the total GSRS, the constipation and abdominal pain GSRS subscale scores with blueberry ingestion. This may in part be attributed to the higher fiber content of the blueberry treatment. The absence of significant treatment differences may be due to a true absence of relevant effects, to similar monosaccharide and polysaccharide components in the blueberry and placebo treatments, or to an insufficient sample size. Effects of blueberry ingestion on stool consistency, GI motility or sensation have not been reported previously in either healthy or FGID subjects to the best of our knowledge. The sample size was calculated to show a clinically significant change in GSRS and was greater than many comparative trials. Nonetheless, the effect of placebo on some of the outcome variables may have been underestimated.

Blueberries can via its polyphenol and sugar components modulate several mechanisms implicated in the pathogenesis of FGID, including inflammation, neural sensitization, intestinal permeability, and the composition and metabolism of the enteric microbiota [3,11,12,13,35,36,37,38,39]. A recent meta-analysis of polyphenol effects on the enteric microbiota showed significant modulation by blueberry powder in eight human studies of similar duration as the current study [39]. Both the sugar-acid fraction of blueberries and maltodextrin, as used in the placebo preparation, can also modulate the composition and activity of the enteric microbiota, especially when ingested without the fruit fiber matrix [40,41]. Further distinction of the effects of the different components of blueberries on GI and extra-GI function need to be performed in future studies.

The standardized fructose breath test was included in the study to investigate the role symptoms and fermentation induced by fructose in FGID and their modulation by blueberries. The gaseous metabolites formed in the intestine are dependent on the characteristics of the intestinal microbiota and are partially absorbed from the intestine, exhaled, and captured during breath testing [42,43]. Fructose intolerance is common in FGID and is probably due to abnormal fermentation by the microbiota accompanied by intestinal hypersensitivity [5,44]. As the polyphenol, fiber, and sugar components of the study treatments exert effects on the enteric microbiota, changes in breath gas concentrations on fructose challenge would be expected. However, the breath tests may be inadequately sensitive and/or specific to demonstrate differences between the treatments. The significant carry-over effect observed for methane production after fructose challenge indicates a prolonged change in the metabolism or composition of methane-producing archaea in both the blueberry and placebo treatments that outlasted the duration of the washout period. Fecal microbiota analysis taking baseline composition into consideration will likely yield more differentiated results.

The improved overall functioning, performance, and quality of life (OQ45.2 scores) may also be related to a reduction in the extra-GI symptoms that are prevalent in patients with FGID [5]. Several recent reviews have summarized the effects of blueberries or polyphenols in various formulations on musculoskeletal and cognitive function in different human cohorts, but effects in FGID have so far not been reported [13,15,45,46,47].

### Limitations

Study limitations have been discussed in the text above. Technical issues are challenging in prolonged cross-over supplementation studies. As a reasonable compromise we chose freeze-dried whole blueberries of the same varieties, a color-, taste-, sugar- and energy-matched placebo used in many previous blueberry studies, a Swiss population with an exceptionally high dosing compliance rate of around 95% and high awareness of the necessity of maintaining stable background dietary habits, and dosing and wash-out periods recommended in IBS and dietary studies. Reproduction of these results in other settings across a wider range of berry doses is encouraged.

The statistical methodology employed conforms with recent guidelines for cross-over trials, including the evaluation of period and carry-over effects and the avoidance of baseline comparisons [48].

## 5. Conclusions

General well-being, quality of life, and life functioning, as well as abdominal symptom relief were improved significantly by blueberry ingestion compared to placebo in patients with FGID. The polyphenol and fiber components of blueberries appear to exert broad beneficial effects separate from any sugar effects implicit to both treatments. There were no significant differences in treatment effects on more specific markers of GI function. In future studies a detailed separation of the polyphenol, fiber, and sugar effects on the mechanisms implicated in FGID would be helpful to provide clinical treatment guidelines.

## Figures and Tables

**Table 1:** Patient characteristics.

	Sequence Placebo/Blueberry n = 23	Sequence Blueberry/Placebo n = 20	*p*-Value of Comparison
Female, n (%)	20 (87)	17 (85)	1.00
Male, n (%)	3 (13)	3 (15)	1.00
Age, years ^ǂ^	31.3 ± 11.6	30.8 ± 9.0	0.87
BMI (kg/m^2^) ^ǂ^	23.6 ± 4.2	21.9 ± 3.2	0.14
IBS, n (%) *	20 (87)	16 (80)	0.69
FD, n (%) *	18 (78)	16 (80)	1.00
Smoker, n (%)	3 (13)	2 (10)	1.00
Food preference, n (%)			
-Omnivore	15 (65)	15 (75)	0.75
-Vegetarian	7 (30)	4 (20)	
-Vegan	1 (4)	1 (5)	
STAI-S ^ǂ^	47.1 ± 3.6	46.2 ± 4.6	0.44
STAI-T ^ǂ^	45.5 ± 6.4	46.5 ± 5.8	0.62
HADS depression score ^ǂ^	10.3 ± 10.0	9.4 ± 5.1	0.70
PHQ-15 ^ǂ^	5.3 ± 4.8	4.8 ± 2.8	0.68
IPHQ, n (%)			
-low	5 (23)	3 (15)	0.70
-moderate	16 (73)	17 (85)	
-high	1 (5)	0	

^ǂ^ mean ± SD * IBS and FD prevalence exceeds 100% due to overlap, FGID: Functional gastrointestinal disorders; IBS: Irritable bowel syndrome; FD: Functional dyspepsia; STAI-S/T: State-Trait Anxiety Inventory, -S: State, -T: Trait; HADS: Hospital Anxiety and Depression Scale; PHQ-15: Patient Health Questionnaire-15; IPAQ: International Physical Activity Questionnaire.

**Table 2:** Treatment effects after blueberry and placebo treatment for six weeks.

Outcome Variable	Sequence	n	Value after 6 Weeks’ Treatment Means ± SD		Blueberry vs. Placebo Means (95% CI)	P_treatment_	P_carry-over_	P_period_
			Period 1	Period 2				
Overall GI symptoms GSRS total	Placebo/Blueberry	23	2.7 ± 1.0	2.3 ± 0.9	−0.3 (−0.6 to 0.0)	0.09	0.15	0.32
	Blueberry/Placebo	20	2.8 ± 1.0	2.9 ± 0.9				
Bloating GSRS	Placebo/Blueberry	23	3.3 ± 1.3	3.1 ± 1.5	−0.2 (−0.7 to 0.3)	0.49	0.13	0.80
	Blueberry/Placebo	20	3.6 ± 1.5	3.9 ± 1.4				
Diarrhea GSRS	Placebo/Blueberry	23	2.4 ± 1.5	1.8 ± 0.9	−0.2 (−0.7 to 0.2)	0.34	0.26	0.08
	Blueberry/Placebo	20	1.9 ± 0.8	1.7 ± 1.0				
Constipation GSRS	Placebo/Blueberry	23	2.3 ± 1.1	1.9 ± 0.8	−0.3 (−0.7 to 0.2)	0.19	0.06	0.68
	Blueberry/Placebo	20	2.5 ± 1.1	2.7 ± 1.3				
Abdominal pain GSRS	Placebo/Blueberry	23	2.9 ± 1.3	2.5 ±1.2	−0.3 (−0.6 to 0.0)	0.08	0.26	0.18
	Blueberry/Placebo	20	3.1 ± 1.3	3.1 ± 1.3				
Functioning & QOL OQ 45.2	Placebo/Blueberry	23	64.5 ± 13.0	58.9 ± 15.4	−3.2 (−5.6 to −0.7)	0.01	0.13	0.05
	Blueberry/Placebo	20	67.8 ± 12.5	68.4 ± 15.2				
Fructose breath test—GI symptoms (AUC × 2 h)	Placebo/Blueberry	23	2.5 ± 3.1	2.5 ± 3.1	−0.1 (−0.9 to 0.7)	0.85	0.91	0.80
	Blueberry/Placebo	20	2.4 ± 3.1	2.5 ± 3.0				
Fructose breath test—CNS symptoms (AUC × 2 h)	Placebo/Blueberry	23	0.9 ± 1.3	1.2 ± 1.3	0.2 (−0.3 to 0.7)	0.47	0.89	0.47
	Blueberry/Placebo	20	1.1 ± 2.0	1.1 ± 1.7				
Fructose breath test—hydrogen (AUC concentration ppm × 2 h)	Placebo/Blueberry	23	28.0 ± 34.7	25.2 ± 34.5	−6.6 (−15.3 to 2.1)	0.14	0.21	0.38
	Blueberry/Placebo	20	35.3 ± 38.8	45.7 ± 44.5				
Fructose breath test—methane (AUC concentration ppm × 2 h)	Placebo/Blueberry	23	3.3 ± 3.9	3.0 ± 4.9	−1.1 (−2.5 to 0.3)	0.13	0.04	0.25
	Blueberry/Placebo	20	5.1 ± 5.0	7.0 ± 6.4				

AUC: area-under-the-curve, ppm = parts per million; CNS: central nervous system; FGID: Functional gastrointestinal disorders; GSRS: Gastrointestinal Symptom Rating Scale; OQ45.2: Outcome Questionnaire 45.2; QOL: quality of life.

## Data Availability

Requests for sharing of the deidentified participant data should be addressed to CWS. Data sharing is subject to the conditions imposed by the Ethics review board.

## Abbreviations

AUC	area-under-the-curve
DGBI	Disorders of gut–brain interaction
FD	Functional dyspepsia
FGID	Functional gastrointestinal disorders
GI	Gastrointestinal
GSRS	Gastrointestinal Symptom Rating Scale
HADS	Hospital Anxiety and Depression Scale
IBS	Irritable bowel syndrome
IPAQ	International Physical Activity Questionnaire
PHQ-15	Patient Health Questionnaire-15
OQ45.2	Outcome Questionnaire 45.2
STAI-S/T	State-Trait Anxiety Inventory, -S: State, -T: Trait
